# Supplementary figures and images for: A single nucleotide polymorphism in the HOMER1 gene is associated with sleep latency and theta power in sleep electroencephalogram
Source: PLoS One. 2020 Jul 9;15(7):e0223632. doi: 10.1371/journal.pone.0223632 (PMC7347117; doi:10.1371/journal.pone.0223632)

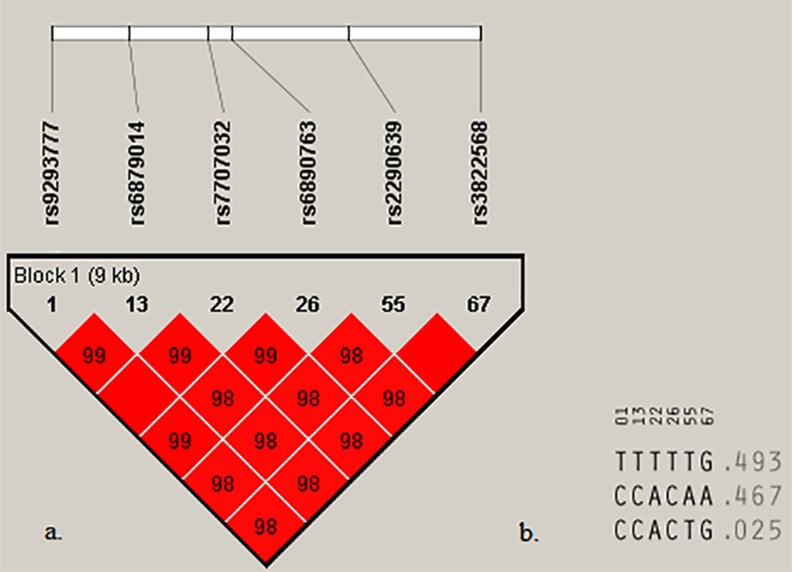

Supplement: S1 Fig — S1A Fig LD plot of six SNPs in HOMER1 gene. Dark red shading denotes D' values (i.e., 99 means D' of 0.99). Squares with no numbers indicates D' of 1. The numbers 1, 13, 22, 26, 55 and 67 in the first line indicate the position of the six strongest (D’ ≥ 0.90) SNP linkage among 67 SNPs in the region. S1B Fig Inferred haplotypes for the LD block with frequencies). HaploView (version 4.2). (TIF) [file pone.0223632.s001.tif]
