# Supplementary material for: A single nucleotide polymorphism in the HOMER1 gene is associated with sleep latency and theta power in sleep electroencephalogram
Source: PLoS One. 2020 Jul 9;15(7):e0223632. doi: 10.1371/journal.pone.0223632 (PMC7347117; doi:10.1371/journal.pone.0223632)
Supplement: Attachment — Submitted filename: Response to Reviewers.pdf [file pone.0223632.s002.pdf]

Editors
PLOS ONE
April 14, 2020

Dear Dr. Romigi,

We thank you and the reviewers for a thorough reading and constructive criticism of our manuscript and for the opportunity to revise and resubmit. We are pleased to submit the improved research article. On the following pages, you will find our response to the editor and reviewer comments.

On behalf of my co-authors, I thank you for your consideration of this resubmission. We appreciate your time and look forward to your response.

Sincerely,

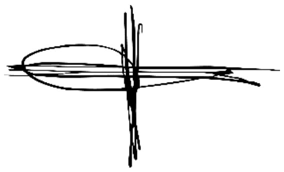

Mario Pedrazzoli, Ph.D. (corresponding author)
pedrazzo@gmail.com, +55 11 3091-8831

PONE-D-19-26778
A single nucleotide polymorphism in the HOMER1 gene is associated with sleep latency and theta power in sleep electroencephalogram
PLOS ONE

Dear Prof Pedrazzoli,

Thank you for submitting your manuscript to PLOS ONE. After careful consideration, we feel that it has merit but does not fully meet PLOS ONE's publication criteria as it currently stands. Therefore, we invite you to submit a revised version of the manuscript that addresses the points raised during the review process.

We would appreciate receiving your revised manuscript by Apr 17 2020 11:59PM. When you are ready to submit your revision, log on to https://www.editorialmanager.com/pone/ and select the 'Submissions Needing Revision' folder to locate your manuscript file.

If you would like to make changes to your financial disclosure, please include your updated statement in your cover letter.

To enhance the reproducibility of your results, we recommend that if applicable you deposit your laboratory protocols in protocols.io, where a protocol can be assigned its own identifier (DOI) such that it can be cited independently in the future. For instructions see: http://journals.plos.org/plosone/s/submission-guidelines#loc-laboratory-protocols

The majority of our protocols involve standard methods such as the scores of polysomnography data and SNP genotyping. We have specified the thermal conditions in Material and Methods section ("rs3822568 Genotyping" subsection). We do not think it is necessary to specify other conventional protocols involved in our study.

Please include the following items when submitting your revised manuscript:

- *A rebuttal letter that responds to each point raised by the academic editor and reviewer(s). This letter should be uploaded as separate file and labeled 'Response to Reviewers'.*
- *A marked-up copy of your manuscript that highlights changes made to the original version. This file should be uploaded as separate file and labeled 'Revised Manuscript with Track Changes'.*
- *An unmarked version of your revised paper without tracked changes. This file should be uploaded as separate file and labeled 'Manuscript'.*

Please note while forming your response, if your article is accepted, you may have the opportunity to make the peer review history publicly available. The record will include editor decision letters (with reviews) and your responses to reviewer comments. If eligible, we will contact you to opt in or out.

We look forward to receiving your revised manuscript.

Kind regards,
Andrea Romigi, M.D., Ph.D
Academic Editor
PLOS ONE

Journal Requirements:

1. *When submitting your revision, we need you to address these additional requirements.*

Please ensure that your manuscript meets PLOS ONE's style requirements, including those for file naming. The PLOS ONE style templates can be found at http://www.journals.plos.org/plosone/s/file?id=wjVg/PLOSOne_formatting_sample_main_body.pdf and http://www.journals.plos.org/plosone/s/file?id=ba62/PLOSOne_formatting_sample_title_authors_affiliations.pdf

[Note: HTML markup is below. Please do not edit.]

Reviewers' comments:

Reviewer's Responses to Questions

Comments to the Author

1. Is the manuscript technically sound, and do the data support the conclusions?

The manuscript must describe a technically sound piece of scientific research with data that supports the conclusions. Experiments must have been conducted rigorously, with appropriate controls, replication, and sample sizes. The conclusions must be drawn appropriately based on the data presented.

Reviewer #1: Partly

Reviewer #2: Yes

---

2. Has the statistical analysis been performed appropriately and rigorously?

Reviewer #1: Yes

Reviewer #2: Yes

---

3. Have the authors made all data underlying the findings in their manuscript fully available?

The PLOS Data policy requires authors to make all data underlying the findings described in their manuscript fully available without restriction, with rare exception (please refer to the Data Availability Statement in the manuscript PDF file). The data should be provided as part of the manuscript or its supporting information, or deposited to a public repository. For example, in addition to summary statistics, the data points behind means, medians and variance measures should be available. If there are

restrictions on publicly sharing data—e.g. participant privacy or use of data from a third party—those must be specified.

Reviewer #1: Yes

Reviewer #2: Yes

---

4. Is the manuscript presented in an intelligible fashion and written in standard English?

PLOS ONE does not copyedit accepted manuscripts, so the language in submitted articles must be clear, correct, and unambiguous. Any typographical or grammatical errors should be corrected at revision, so please note any specific errors here.

Reviewer #1: Yes

Reviewer #2: Yes

---

5. Review Comments to the Author

Please use the space provided to explain your answers to the questions above. You may also include additional comments for the author, including concerns about dual publication, research ethics, or publication ethics. (Please upload your review as an attachment if it exceeds 20,000 characters)

Reviewer #1: In this study, the authors evaluated the association of a specific SNP in the HOMER1 gene with specific aspects of sleep in a representative sample of the Brazilian population. They in fact hypothesize that, given the importance of the glutamatergic system regulating sleep and prolonged wakefulness, genetic variation in the HOMER1 gene might explain inter-individual variability in several sleep traits measured using polysomnography.

Even if they studied only a gene and only one SNP of this gene, the study is well conducted and the sample size of studied subjects is consistent

The research is quite original, the experiments and the statistical analyses were conducted in a proper way and the paper is written in a clear and intelligible way.

Some minor issues are to be considered:

The author should better discuss that this study involve only one gene (and one SNP) but the phenotypes they are discussing is multifactorial and the genetic factors involved can be numerous. The gene they are studying may give a contribution but it does not explain the whole genetic variability of these phenotypes.

Thank you for pointing it out. We have added a paragraph (L229-234) in Discussion section arguing about this matter:

“When considering that the relationship between disease susceptibility and genetic variation is complex, and for the most phenotypes more than one gene is generally involved, care has to be taken when considering just one SNP in one gene to draw conclusions about a complex and multifactorial phenotype as sleep. Nevertheless, when considering the physiological mechanisms related to glutamate neurotransmission and previous associations with sleep, *HOMER1* sounds like a good candidate gene for sleep phenotypes related to extended wake like insomnia.”

They should also discuss about the possibility of other SNPs (and HAPLOTYPES) of this gene involved Are already known other SNPs? Could they sequence the entire gene in a group of subjects in order to study this variability and the existence of other SNPs? They should at least discuss it.

We appreciate your suggestion. However, it is not possible at this time to sequence the samples. Therefore, we have added a supplemental material informing about the linkage disequilibrium (S1A Figure) and also a paragraph (L235-240) discussing the possibilities for other SNPs in *HOMER1* gene that might have a role in the phenotype:

“*HOMER1* has many single nucleotide polymorphisms along its sequence. According to the 1000 Genomes Project data, in American populations rs3822568 is in strong linkage disequilibrium with at least five other SNPs (S1 Figure), although all of them are localized in intergenic regions and therefore have low potential to yield genetic consequences. Also, neither of them has been associated with any relevant clinical phenotype. This fact does not exclude the possibility of other SNPs and even phased haplotypes play important roles in the genetic control of the sleep parameters analyzed in this study.”

They should also discuss about the functional role and meaning of the SNP they analyzed.

Great point. We have added a new paragraph (L220-L228) in Discussion, which we cautionary suggest **(L226-228)** the role of rs3822568 in a physiological context:

“In the view of recent studies about glutamatergic neurotransmission, sleep homeostasis and synaptic plasticity, *Homer1* is strictly linked to the (mGluR5) function. It selectively uncouples mGluR5 from the effector targets in the postsynaptic density (33) that results in attenuation of mGluR5-mediated activation of phospholipase C and downstream signaling (34). In humans, increased mGluR5 availability after sleep deprivation was tightly associated with increased propensity to fall asleep (35) and considering the interplay between *Homer1* and mGluR5, it has been suggested that *Homer1a* serves as a molecular integrator of arousal and sleep need (36). **In that way, we may cautionary propose the SNP described here could mediates the integration *Homer1*/mGluR5 through 3’UTR mechanisms cited above, and therefore would mediates a compensatory mechanism to promote wakefulness in the sleep-deprived state like insomnia states.**”

Reviewer #2: In the current manuscript Pedrazzoli et al., examine the association between overnight sleep parameters and a polymorphism in the Homer1 gene in a Brazilian population. The authors report significant genotype interactions in the Homer1 3'UTR and sleep latency, efficiency and other parameters, as well as an interaction with Theta power in the EEG recordings. Recent studies have revealed genetic links with the Homer1 gene and psychiatric conditions. Basic research has also shown an important

role for the Homer1 gene in sleep behavior in rodents and insects. The current work is presented in a straight forward manner and the data collection has been done rigorously and appropriately.

Considering the material covered in this manuscript it would be highly appropriate to include in the bibliography a recent high profile paper on the role of Homer1a in synaptic plasticity during sleep. (Diering et al., 2017 Science, PMID: 28154077). This paper shows that Homer1a protein is important to regulate mGluR1/5 signaling during sleep and for sleep-dependent remodeling of synapses. A second paper Holst et al., 2017 eLife, PMID: 28980941 shows that mGluR5 is upregulated during sleep deprivation in humans and is required for appropriate response to sleep deprivation in mice.

I have no other concerns about the current manuscript.

Thank you for the suggestion. We have added a new paragraph (L220-L228) in Discussion with references suggested above and other:

"In the view of recent studies about glutamatergic neurotransmission, sleep homeostasis and synaptic plasticity, *Homer1* is strictly linked to the (mGluR5) function. It selectively uncouples mGluR5 from the effector targets in the postsynaptic density **(33)** that results in attenuation of mGluR5-mediated activation of phospholipase C and downstream signaling **(34)**. In humans, increased mGluR5 availability after sleep deprivation was tightly associated with increased propensity to fall asleep **(35)** and considering the interplay between *Homer1* and mGluR5, it has been suggested that *Homer1a* serves as a molecular integrator of arousal and sleep need **(36)**. In that way, we may cautionary propose the SNP described here could mediates the integration *Homer1*/mGluR5 through 3'UTR mechanisms cited above, and therefore would mediates a compensatory mechanism to promote wakefulness in the sleep-deprived state like insomnia states."

---

6. PLOS authors have the option to publish the peer review history of their article (what does this mean?). If published, this will include your full peer review and any attached files.

If you choose "no", your identity will remain anonymous but your review may still be made public.

Do you want your identity to be public for this peer review? *For information about this choice, including consent withdrawal, please see our Privacy Policy.*

Reviewer #1: No
Reviewer #2: Yes: Graham Hugh Diering

[NOTE: If reviewer comments were submitted as an attachment file, they will be attached to this email and accessible via the submission site. Please log into your account, locate the manuscript record, and check for the action link "View Attachments". If this link does not appear, there are no attachment files to be viewed.]

While revising your submission, please upload your figure files to the Preflight Analysis and Conversion Engine (PACE) digital diagnostic tool, https://pacev2.apexcovantage.com/. PACE helps ensure that figures meet PLOS requirements. To use PACE, you must first register as a user. Registration is free. Then, login and navigate to the UPLOAD tab, where you will find detailed instructions on how to use the tool. If you encounter any issues or have any questions when using PACE, please email us at figures@plos.org. Please note that Supporting Information files do not need this step.
